# Supplementary material for: A Systematic Review and Meta-Analysis of Immunoglobulin G Abnormalities and the Therapeutic Use of Intravenous Immunoglobulins (IVIG) in Autism Spectrum Disorder
Source: J Pers Med. 2021 May 30;11(6):488. doi: 10.3390/jpm11060488 (PMC8229039; doi:10.3390/jpm11060488)
Supplement: Supplementary file 1 [file jpm-11-00488-s001.zip › Supplementary Figure 1.pdf]

PRISMA 2020 flow diagram for new systematic reviews which included searches of databases, registers and other sources

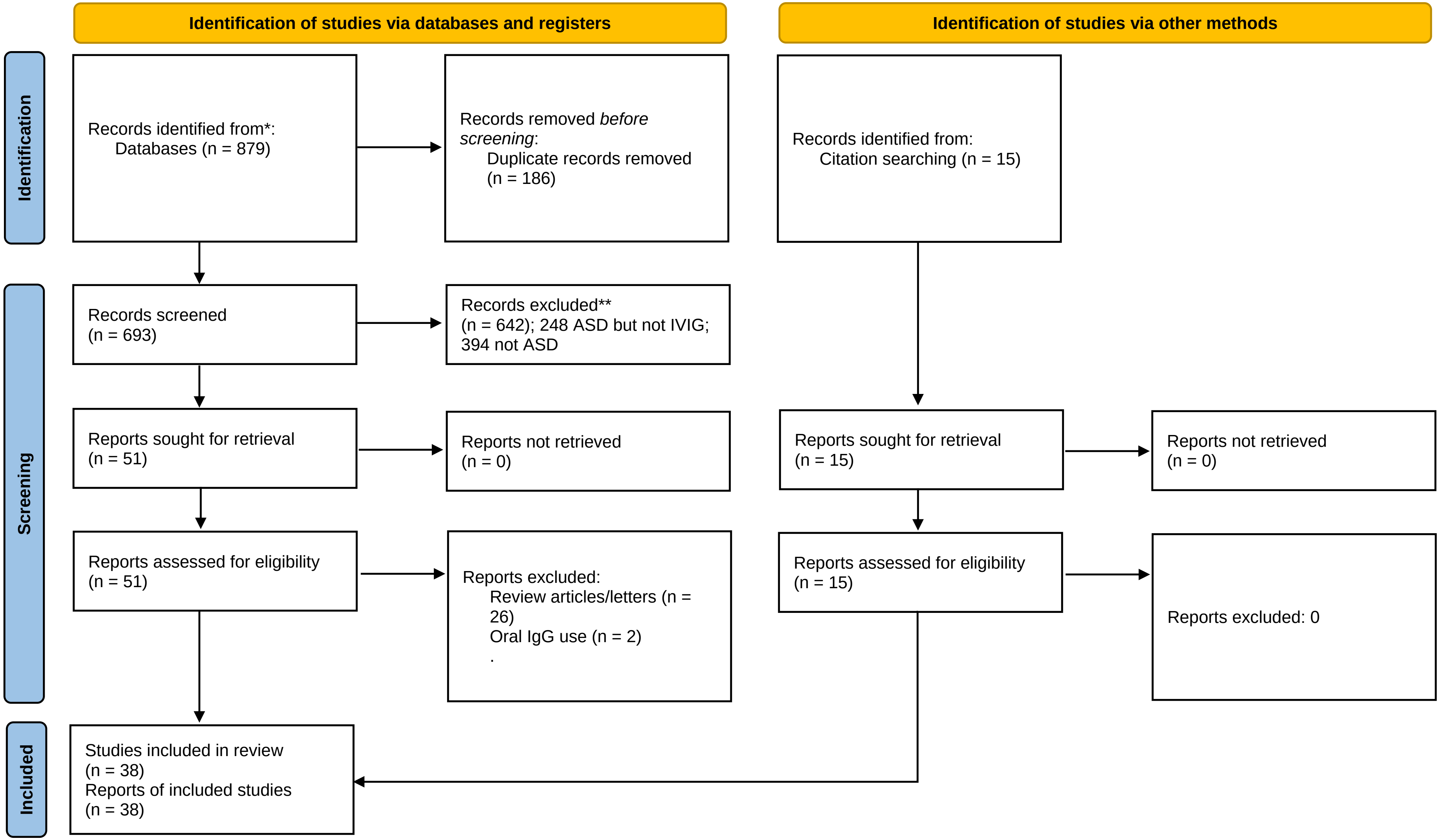

From: Page MJ, McKenzie JE, Bossuyt PM, Boutron I, Hoffmann TC, Mulrow CD, et al. The PRISMA 2020 statement: an updated guideline for reporting systematic reviews. BMJ 2021;372:n71. doi: 10.1136/bmj.n71. For more information, visit: http://www.prisma-statement.org/
