# Supplementary material for: A Systematic Review and Meta-Analysis of Immunoglobulin G Abnormalities and the Therapeutic Use of Intravenous Immunoglobulins (IVIG) in Autism Spectrum Disorder
Source: J Pers Med. 2021 May 30;11(6):488. doi: 10.3390/jpm11060488 (PMC8229039; doi:10.3390/jpm11060488)
Supplement: Supplementary file 1 [file jpm-11-00488-s001.zip › Supplementary Table 2.pdf]

Supplementary Table 2. Number of Participants for Meta-analysis of studies on immunoglobulin G Concentration in Autism Spectrum Disorder.

	Non-Siblings		Siblings		All Controls	
	**ASD**	**Controls**	**ASD**	**Controls**	**ASD**	**Controls**
Total IgG	158	132	65	43	223	175
IgG1	132	118	65	43	197	161
IgG2	132	118	65	43	197	161
IgG3	132	118	65	43	197	161
IgG4	132	118	65	43	197	161
